# A user's guide to your first self-driving liquid handling lab

**DOI:** 10.1039/d5dd00525f

**Published:** 2026-03-25

**Authors:** Apostolos P. Maroulis, Dylan M. Waynor, Quinn M. Gallagher, Roshan A. Patel, Matthew Tamasi, D. Christopher Radford, Michael A. Webb, Adam J. Gormley

**Affiliations:** a Department of Biomedical Engineering, Rutgers, The State University of New Jersey Piscataway NJ 08854 USA adam.gormley@rutgers.edu; b Department of Chemical and Biological Engineering, Princeton University Princeton NJ 08544 USA mawebb@princeton.edu

## Abstract

Experimentation is inherently difficult because most methods require substantial refinement, calibration, and validation before high-quality, reliable data can be collected. In most cases, experimental outcomes are impacted by multiple variables, thus requiring their simultaneous optimization for single and multi-objective targets. Traditional experimental approaches rely on trial-and-error methods guided by rational decision making, but these become increasingly inefficient and ineffective as complex interactions between inputs limit our ability to capture underlying trends using conventional statistical approaches. Machine learning and active learning (ML/AL) combined with automation represents an approach that can bolster future laboratory productivity. However, a steep initial learning curve and high costs of instrumentation pose substantial barriers to adoption. To democratize access, we herein comprehensively cover both the computational skills and hardware implementation necessary for self-driven experimental workflows. The accompanying open-source, low-cost liquid handling platforms offer practical templates for researchers adopting self-driving lab (SDL) methodologies. Complete tutorials and build guides are provided at https://gormleylab.github.io/SDLGuide.

## Abstract 2

To contribute towards the democratization of self-driving laboratories, we created a foundation-level guide focusing on Bayesian optimization driven experimentation and low-cost open-source automated liquid handlers.

## Graphical abstract

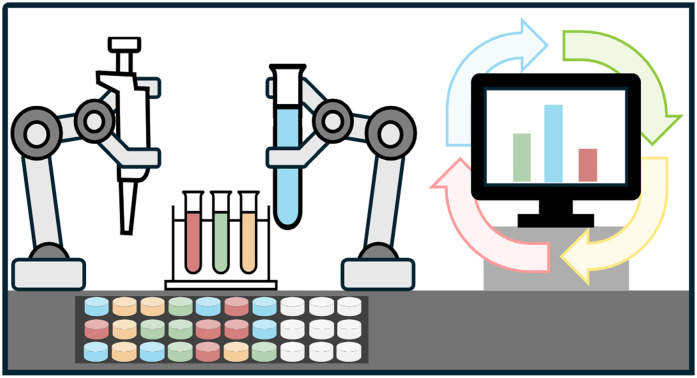

## 1 Introduction

Major goals of scientific research include advancing fundamental understanding of phenomena and developing technologies. In practice, this often involves using experimentation to determine how controllable input parameters (*e.g.*, processing conditions, molecular structure, composition) influence a complex output property of interest (*e.g.*, material performance, yield, selectivity, and stability). Traditionally, researchers have selected experiments by leveraging their own intuition (*e.g.*, rational experimental design) or more systematic approaches such as classical design of experiments (DOE),^1,2^ which are often supplemented by simple statistical methods to model the relationship between experimental parameters and the property of interest.^3^ While powerful, these approaches can face limitations when the input parameter space is high-dimensional, the relationship between input parameters and output property is highly nonlinear, and experiments are time and/or resource-intensive.

Recent advances in machine learning (ML) and automation offer new ways to overcome these barriers and accelerate scientific research.^4–6^ Specifically, supervised ML algorithms can provide flexible modeling frameworks to predict complex output properties from input parameters with unprecedented fidelity.^7–9^ When systematically probed and interpreted, supervised ML algorithms can yield insights into how input parameters and their interactions influence output properties.^10–12^ In addition, active learning (AL) algorithms can be used to strategically select the ‘next best experiment(s)’, typically to improve model fidelity (thus bolstering knowledge) or to optimize output properties with respect to input parameters.^12–16^ Complementing these algorithmic advances, robotic platforms and automated high-throughput characterization tools allow experiments to be performed rapidly, reproducibly, and in parallel, in effect, providing more data to resolve the connection between input parameters and output properties in a shorter period of time.^17–19^ Furthermore, data collected when applying these tools allow for greater repeatability of experimentation through the reduction of human error when generating samples. Thus, mitigating uncontrolled variations captured by the model and when capturing experimental baselines.

**Fig. 1:**
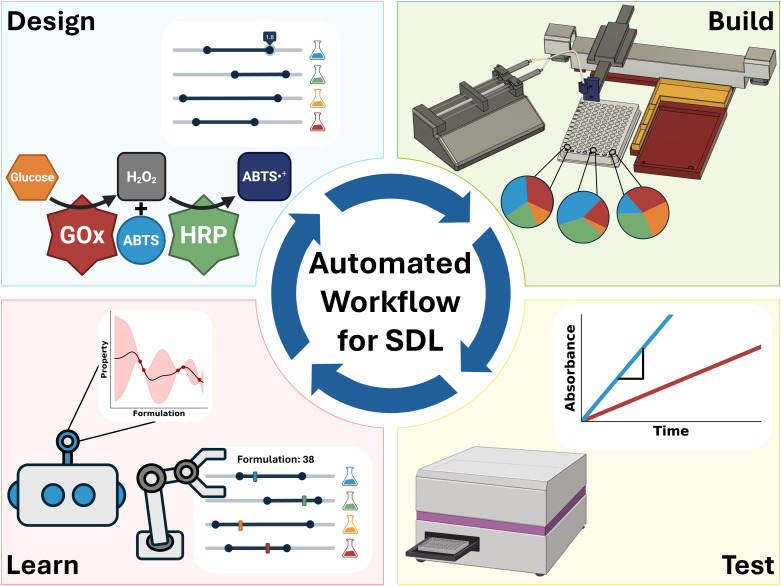
Schematic of a self-driving laboratory (SDL) workflow based on a closed-loop design-build-test-learn paradigm for automated experimentation. Experiments are autonomously performed using a custom and low-cost liquid handler. The collected data, alongside design parameters, are fed into a machine learning pipeline that guides data acquisition with active learning principles. This framework can be used to understand system behavior across an input domain or to autonomously suggest and test parameter combinations to yield desired functionality.

Together, the aforementioned components can be linked together in a Design-Build-Test-Learn (DBTL) workflow,^20^ as illustrated in Fig. 1. In a DBTL workflow, high throughput/automated machinery expedite the experimental preparation (build) and characterization (test) of samples, supervised ML enables modeling and projection of the collected data (learn), and AL leverages the learned information to select new experiments (design). This process can be repeated towards the advancement of defined scientific objectives. If well-integrated, such that the process can sustain without human intervention, the assembly of these components in a DBTL workflow forms a self-driving lab (SDL).^6,21^ Distinguished by rapid and efficient operation, SDLs have the potential to revolutionize scientific discovery and otherwise influence how scientific research is conducted.

Despite their potential, implementing automated DBTL workflows can present substantial challenges that add methodological complexity with minimal immediate gain. Currently, the time-investment and expansive skill set required—which can span programming, robotics, and domain expertise—often drives researchers toward seeking commercial solutions for their bespoke objectives. Commercial turn-key systems offer reliable, out-of-the-box operation that alleviates these problems, which can be attractive to a user with sufficient capital. However, direct investment may be improbable for a researcher due to high initial cost. Companies, such as Opentrons, have helped lower entry barriers with more accessible (*i.e.*, low-cost and open-source) turn-key instruments,^22^ but the investment in compatible devices and infrastructure still may exceed that of many research laboratories.^23^ This creates a critical gap between the promise of SDL methodologies and their practical adoption in typical research settings.

To address aforementioned barriers related to skills and resources, there are growing efforts to democratize access to SDLs through open-source software and hardware.^22,24^ For example, the Jubilee Project's extensible multi-tool motion platform demonstrates this idea through community built open-source hardware for automation applications.^23^ Additionally, previous examples of low-cost open-source tools for prototyping SDLs (*e.g.*, SDL-light and claude-light) illustrate more community-driven approaches for automated experimentation.^25,26^ In particular, wet lab SDLs based on liquid handling devices have demonstrated success across diverse applications.^6,27–32^ Nevertheless, a significant gap remains between these demonstrations and the step-by-step educational resources that researchers need to successfully and timely implement SDL workflows.

Herein, we address both an educational and infrastructure gap by presenting a comprehensive guide for building and implementing an open-source, low-cost liquid handling platform capable of SDL applications. Our approach combines hardware construction with software development, featuring Python-based control scripts and tutorials that researchers can customize for their specific needs. This provides a resource to bolster familiarity with AL/SDL concepts as well as a practical development tool, guiding users through the complete process from hardware assembly to crafting autonomous workflows. Additionally, we believe that the time investment and technical expertise gained through the utilization of our guides will embolden researchers to then tailor learned techniques towards their specific domain. This work represents the second installment for our User's Guide Series, building upon the foundational ML concepts covered in our first installment;^33^ readers are referred to that installment for essential prerequisite knowledge and terminology. Through explanations of both theoretical principles and practical implementation steps, this guide aims to lower barriers for widespread adoption of SDL workflows across diverse research disciplines.

The rest of the document is structured as follows. Section 2 reviews essential ML/AL terminology and requisite background knowledge needed for understanding the guide. Section 3 provides instructional information for the creation and operation of low-cost liquid handlers for the purpose of automation. Section 4 provides a simple yet non-trivial demonstration of an autonomous experiment optimizing an enzyme assay, incorporating the developed liquid handling device and covered topics. Finally, Section 5 summarizes the key topics featured in this guide and provides perspective for future consideration. We note that supporting resources, including complete tutorials and build guides, are available at https://gormleylab.github.io/SDLGuide.

## 2 Essentials of active learning

### 2.1 Overview of active learning and Bayesian optimization

For an SDL to be self-driving, it must have the ability to autonomously propose and then pursue new experiments. The autonomous proposition can be accomplished using AL, which provides a principled framework based on ML and information theory to select experiments judiciously based on previously acquired data. In previous work, AL has been successfully used to develop accurate ML models with minimal training data or optimize outputs from a large input space using minimal evaluations.^12,34–37^ Thus, by implementing this process to function iteratively, the SDL can efficiently acquire data without human intervention.

**Fig. 2:**
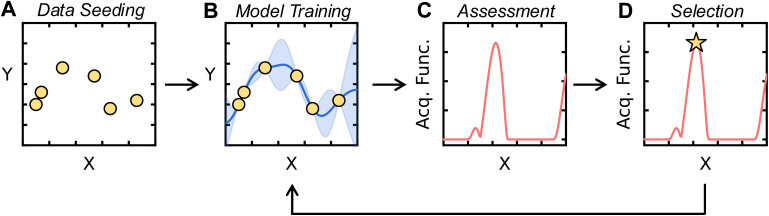
Schematic of active learning framework. (A) Data are initially chosen using a data seeding method. Here, *X* is the range of inputs, *Y* are the property values, and the initial data are plotted as yellow markers. (B) A surrogate model is trained on this data and used to make predictions on the input space. The mean predictions of the surrogate model are plotted as a blue line, while the blue shaded region depicts the uncertainty of the surrogate model. (C) An acquisition function is computed from these predictions and uncertainties. Here, the acquisition function is plotted as a red line over the input space *X*. (D) The point which maximizes the acquisition function is chosen for further evaluation, as shown by the yellow star. After evaluating the new point, the surrogate model is retrained on the new dataset, completing the active learning loop.

Applications of AL for SDLs follow a standard workflow, the components of which will be further described in subsequent sections. A schematic of this workflow is shown in Fig. 2. Initially, high-throughput preparation and characterization capabilities are used to perform a first set of measurements with conditions randomly selected or using another strategy. We refer to this initial data selection as data seeding. An ML model, termed the surrogate model, is trained on this data for target property prediction. The surrogate model then predicts the target property on the unmeasured portions of the input space. Then, new points are selected based on the predictions of the surrogate model. Specifically, an acquisition function is defined, typically using the outputs of the surrogate model, and new points are selected by optimizing the acquisition function. The SDL then obtains data for the recommended points, after which the surrogate model is retrained and the process repeats. This AL “loop” continues until an end criterion is met. Common criterion include exhausting an experimental resource or time budget, the plateauing of surrogate model accuracy, or the identification of a set of experimental conditions that yield a target property.

This standard AL workflow is commonly employed in two distinct contexts. First, AL can be used to select samples that best represent the variation of a property across the input space. In other words, AL is used to train a maximally accurate surrogate model for the prediction of the target property for any possible input. In this context, the acquisition function may rely on the uncertainty of the surrogate model. ML models can make predictions with uncertainty using a variety of the methods discussed in Section 2.3. By choosing points for which the surrogate model is maximally uncertain, the SDL minimizes uncertainty in model predictions across the input space, resulting in a (hopefully) maximally accurate surrogate model.

Second, AL can be used to find points in an SDL's input space that optimize a target property using minimal experiments. This form of AL is often referred to as Bayesian optimization (BO). In this context, the acquisition function considers surrogate model predictions and uncertainties to select new points from the input space which are likely to improve over the current optimal input. Since SDLs are often employed for optimizing experimental conditions or discovering materials with optimal properties, BO is commonly used to guide autonomous data selection in SDLs. Therefore, we emphasize the use of BO in this User's Guide.

For the remainder of Section 2, we provide a more thorough discussion of the components of AL/BO. To provide readers with a hands-on demonstration of the methods and workflow described here, we have created a Google Colab notebook for performing BO on a toy function. Specifically, we show how BO can be applied in various ways to the optimization of the Müller–Brown potential, a two-dimensional function from theoretical chemistry commonly used to benchmark optimization protocols. All code required to recreate the results of Section 2 is available in the Google Colab.

### 2.2 Data seeding

**Fig. 3:**
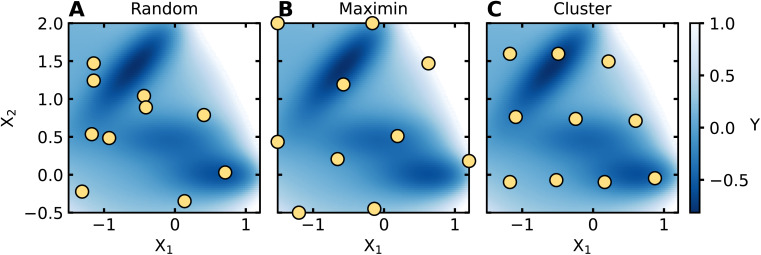
Examples of space-filling algorithms. Common space-filling algorithms are applied to the two-dimensional Müller–Brown dataset. *X*_1_ and *X*_2_ are the features of the Müller–Brown potential, and the labels are shown using a blue color map, where darker regions denote smaller values. The chosen sample is shown by yellow points. The results of (A) random, (B) maximin, and (C) cluster-based sampling algorithms applied to the Müller–Brown dataset are shown.

Choosing a set of data to initiate AL/BO is called data seeding. The initial dataset may have been previously collected, or it may be obtained intentionally at the outset of an AL-guided campaign. If the latter, it is desirable to use a diverse set of initial points that can supply baseline coverage across the input space. To generate such diverse initial datasets, space-filling algorithms can be employed;^38^ examples include Latin hypercube sampling (LHS)^39^ and Sobol sampling^40^ when variables on the domain are independent and bounded, while maximin^41^ and cluster-based^42^ samples can be employed for arbitrarily shaped domains. While many options exist, so long as the initial training set covers the input domain, most reasonable options should suffice, as the importance of initial training data selection is expected to decline as more data is acquired during AL.^37^Fig. 3 shows examples of popular data seeding algorithms applied to the Müller–Brown potential, as shown in the Google Colab.

In AL/BO workflows, it is necessary to decide what fraction of an experimental budget should be allocated to data seeding. There is no consensus on the best amount of data to use for seeding, and the ideal amount is likely problem dependent. As a result, seed dataset sizes are usually chosen based on data acquisition logistics. If data acquisition occurs sequentially, as is often the case for manual experimentation, the seed dataset could be a single measurement. If data acquisition occurs in batches, as is often the case for high-throughput experimental equipment, then a seed dataset size can be chosen for compatibility with experimental protocols (*e.g.*, a size of 96 for experiments conducted in 96-well plates). If one has the capacity to choose a seed data size independent of equipment constraints, then it can be decided by considering the AL/BO algorithm being used. If an exploitative BO algorithm is employed, it may be beneficial to allocate a larger portion of one's experimental budget to space-filling, suggesting a larger seed dataset. If a BO algorithm has an exploratory component, it may be beneficial to provide the algorithm more iterations for optimization, suggesting a smaller seed dataset. We emphasize that these are general considerations and that the relationship between seed data and AL/BO outcomes is not fully understood.^19^

### 2.3 Surrogate model training

**Fig. 4:**
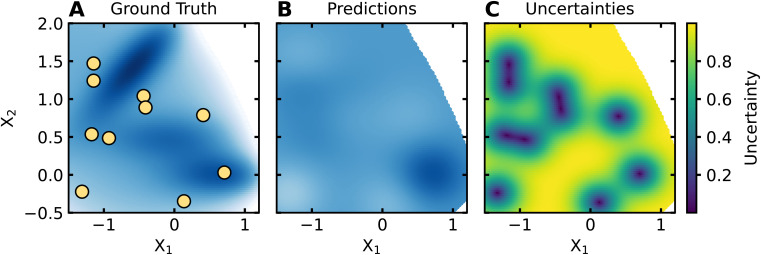
Example of surrogate model outputs. A GP is trained on the dataset shown in (A). The predictions of this GP on the Müller–Brown domain are shown in (B), while the uncertainties of the GP on the Müller–Brown domain are shown in (C). The GP accurately predicts a local minimum in the bottom right corner of the domain. The GP has minimal uncertainty near the training data, but has high uncertainty far from the training data.

Using the initial dataset, an ML model is trained and used to make predictions across the entire input space. While any ML model can be used for AL/BO, some common examples include Gaussian processes (GPs), random forests, and neural networks. GPs are the usual method employed for BO. For most AL/BO workflows, surrogate models are used to make predictions with uncertainty (*i.e.*, quantifying a range of possible predictions), since surrogate model uncertainty is often used to guide the selection of new experiments. Fig. 4 shows a GP's predictions and uncertainties for the Müller–Brown dataset after being trained on the seed dataset shown in Fig. 3A.

ML models differ in how they compute uncertainties, which can impact AL/BO performance. GPs inherently measure uncertainty by predicting distributions of labels directly, which encourages their use in AL/BO workflows. Random forests, being ensembles of decision trees, typically compute uncertainty by measuring the spread in predictions from individual decision trees. For neural networks, uncertainty is often calculated by training several neural networks with different initial parameters in a process called ensembling. In principle, all ML models are capable of uncertainty estimation using ensembling or bootstrap aggregation, where uncertainties are calculated using the spread of predictions from several models trained on different subsets of the training data. Uncertainty quantification for ML models of varying architectures is an active area of research, and we refer interested readers to more complete treatments of uncertainty estimation.^43–47^

### 2.4 Acquiring new data

After the ML model is trained, new points in the input space are selected for evaluation based on the predictions and uncertainties of the ML model. A new candidate is selected if it maximizes an acquisition function. Acquisition functions usually depend on both predicted values and uncertainties of surrogate models. If researchers are interested in maximizing ML model accuracy across the domain, a suitable acquisition function may simply be the model uncertainty. In other scenarios, researchers are interested in finding the experimental inputs that maximize a property; numerous acquisition functions comport with this objective, with opportunity to balance both predicted values and uncertainties when choosing the next candidate from the domain. Researchers may also be interested in simultaneously recommending several points (*i.e.*, batch selection) rather than one point at a time (*i.e.*, sequential selection),^48^ and acquisition functions can be devised or adapted for either context.

**Fig. 5:**
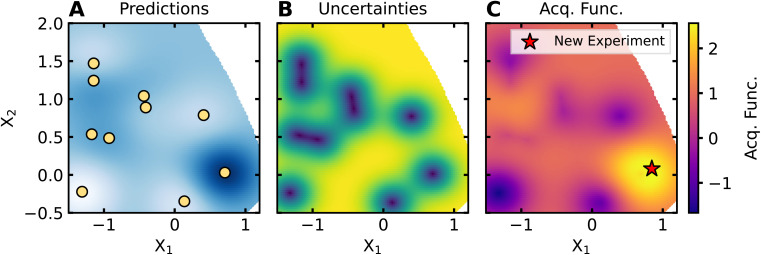
Example of an acquisition function. The expected improvement acquisition function is computed for the predictions and uncertainties of the surrogate model. (A) The scaled predictions of the surrogate model. (B) The uncertainties of the surrogate model. (C) The expected improvement acquisition function. The proposed point which maximizes the acquisition function is shown as a red star.

Numerous acquisition functions are used for BO. Fig. 5 shows the expected improvement (EI) acquisition function calculated for the predictions and uncertainties on the Müller–Brown domain shown in Fig. 4 and the point which maximizes it. The most common acquisition functions for BO include EI, probability of improvement (PI), and upper confidence bound (UCB). PI calculates the probability that a candidate will improve upon the best current result (sometimes referred to as the incumbent). EI extends this concept by also weighting probability of improvement by the magnitude of expected improvement; thus, the consideration evolves from asking simply whether something will be better to how much better it might be. UCB is calculated as a weighted sum of the prediction and uncertainty, thereby choosing points based on their likely maximum value. Variations on these acquisition functions can be formulated with a hyperparameter that modulates the balance of exploitation (*i.e.*, choosing points with predicted optimal values) or exploration (*i.e.*, choosing points with high uncertainty). Additional acquisition functions exist based on different considerations (*e.g.*, entropy search, knowledge gradient, Thompson sampling, *etc.*).^49^ Likewise, ensembles of acquisition functions can be used to combine the intuitions of each approach; this has proven especially useful in batch selection settings.^50,51^

High-throughput experimental equipment developed for SDLs is often capable of making measurements in batches. Therefore, it is useful for AL/BO algorithms to recommend batches of points rather than individual measurements for data acquisition. A naïve approach to select batches of *b* measurements involves choosing the *b* measurements that optimize an acquisition function (*e.g.*, the *b* points with the highest EI). However, this approach does not consider correlation among proposed measurements, possibly leading to redundancy in acquired data. Alternative batch selection methods attempt to choose desirable measurements while reducing redundancy. Some methods sequentially choose batches of points while discouraging selection near previously chosen points, such as local penalization^52^ and Kriging believer batch selection.^48^ Other methods rely on stochasticity to prevent similar measurement selection, like Thompson sampling and the multipoint probability of optimality.^53^ Batch selection methods can also use filters, such as clustering algorithms^54^ or diversity metrics,^55^ to limit redundancy in the selected batch. Batch selection in AL/BO workflows is still an active area of research, and no single approach has proven optimal across a wide array of optimization problems.

### 2.5 Closing the loop

After a new point is recommended, the SDL prepares the necessary experiment, measures the result, and the process is repeated. Specifically, the ML model is retrained on the updated set of measurements, predictions and uncertainties are made on the domain, and the point that maximizes the updated acquisition function is chosen and measured by the SDL. This process is repeated until a stopping criterion is reached, whether that is exhaustion of the SDL budget, identification of an effective input, or sufficient convergence of model accuracy (as suitably measured by cross-validation or a held-out test set).

### 2.6 Successful applications of AL/BO for SDLs

The growing popularity of AL/BO for SDLs is supported by many successes. Tamasi *et al.* used BO (specifically, a GP with the EI acquisition function) to discover copolymers capable of successfully stabilizing three enzymes under thermal stress by measuring only ∼0.1% of the total domain.^12^ Angello *et al.* used BO (specifically, a novel BO formulation called Gryffin^56^) to not only optimize the photostability of light-harvesting donor–acceptor molecules in solution sampling only 1.5% of the total domain, but they also gained chemical “knowledge” by identifying previously unknown molecular features correlated with their target property during their BO campaign.^57^ Szymanski *et al.* used an AL-driven SDL to explore the domain of synthetically accessible inorganic powders, integrating computation, theory, AL, and robotics to propose synthesis recipes and search for novel compounds.^31^ These are just a few examples of a growing effort to use AL/BO and SDLs to accelerate scientific discovery.^58–62^ We anticipate that continued research into maximally data-efficient AL/BO protocols will only further increase the successes of SDLs.

## 3 An open-source SDL platform build

**Fig. 6:**
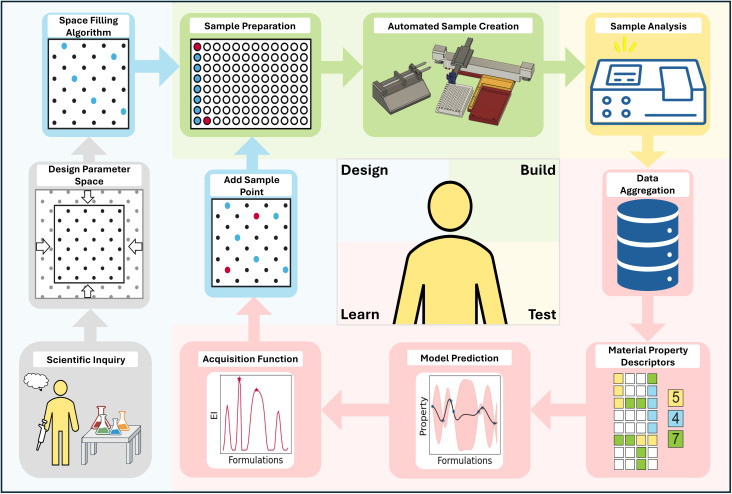
Schematic overview of an SDL. Once an experimental question is generated and the researcher designs a parameter space of relevant resources, the task is sent through the automated platform to iteratively uncover the function–property relationships in the domain. The automated platform uses a designated space filling algorithm, prepares and analyzes chosen samples, aggregates the data into a ML friendly format, and performs BO by applying a chosen ML model and acquisition function to suggest the next samples.

While Section 2 introduced readers to the essential facets of AL, the goal of this section is to deploy these methods in a simple, low-cost, and open-source platform for self-driving wet lab experimentation. Fig. 6 shows how the principles of AL/BO can be incorporated into an automatic experimental platform to achieve this goal. We view our platform as an accessible and customizable alternative to commercial devices such as the Opentrons OT-2 robot. Inspired by SDL-Light and Jubilee,^23,25^ we provide an SDL build guide that provides two lower cost and open-source alternatives to commercially available liquid handlers. By developing this guide, we also hope to encourage the use of automatic liquid handlers in experimental workflows and provide a starting point for the iterative refinement of low-cost, automated laboratory equipment.

### 3.1 Custom-built liquid handlers

**Fig. 7:**
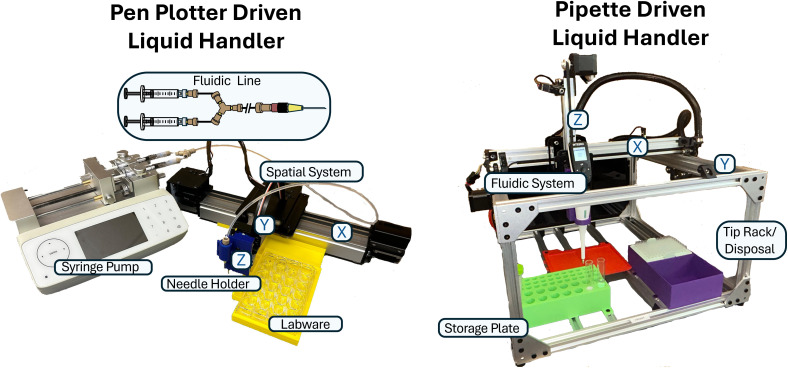
Completed examples of compatible low-cost liquid handler platforms for an SDL (pen plotter driven system described in length in this paper). Includes both fluidic schematics, and spatial movement systems. The pen plotter driven liquid handler's spatial system is comprised of a modified pen plotter while the fluidic system is comprised of a user-developed fluidic line driven by a controllable syringe pump. The pipette driven liquid handler's spatial system is comprised of a user-developed gantry while the fluidic system is comprised of a wirelessly controlled electronic pipette. Build guides for both liquid handlers including parts lists, CAD files, and software can be found in GitHub at https://gormleylab.github.io/SDLGuide.

Automated liquid handlers rely on three systems: the spatial system, fluidic system, and control system. The spatial system includes mechanical parts capable of movement, the fluidic system includes equipment capable of drawing and dispensing fluid, and the control system includes the software necessary to coordinate the spatial and fluidic systems to execute user-defined tasks. Based on these systems, we prototyped two liquid handling platforms which offer lower cost alternatives to commercial instruments. Fig. 7 shows these platforms, referred to as the pen plotter- and pipette-driven liquid handlers, which differ in their spatial and fluidic systems. The platforms strike a balance between dispensing accuracy, precision, and speed, while being built for less than $1000 (excluding the cost of the syringe pump, electronic pipette, and UV-vis spectrophotometer, which are common in most labs).

The pen plotter driven liquid handler was created by integrating a Fusion 200X syringe pump by ChemyX Inc., an AxiDraw V3 pen plotter by Evil Mad Scientist (now sold by Bantam Tools as NextDraw), various fluidic components obtained from Chrom-Tech, a supplier of IDEX Health and Science fluidic components, and 3D-printed parts designed and printed using a Bambu Lab X1C 3D printer. The pen plotter was used as a controllable, three-axis, pre-built spatial system for precise movement. The Fusion 200X syringe pump was chosen as the basis of our fluidic system for its precision bulk withdrawal and infusion at large volumes. Due to the commercial availability and well documented APIs of the discussed devices, it is simple to build a programmable control system. The platform is designed to work with a SpectraMax M2 UV-vis spectrophotometer plate reader (*i.e.*, from Molecular Devices Inc.) to collect absorbance data from microplates, as can be seen in Fig. S2. A 3D printed platform is designed to fit auxiliary labware such as reagent holders, waste, and cleaning solution with designated space opening and closing the plate reader drawer. This allows for transfer of materials from the reagent holders to the sampling plate inserted into the plate reader. A demonstration of this system running can be seen in Video S1.

To provide an alternative to the pen plotter-driven liquid handler that may serve different needs within a laboratory environment, we provide a build guide for a pipette-driven liquid handler. Here, the fluidic system is comprised solely of an electronic pipette (VIAFLO, Integra Biosciences) which individually withdraws and dispenses in small volumes with the commercially determined precision. The fluidic system is mounted on a custom gantry that acts as the spatial system, as shown in Fig. 7. With carriages along the *X*/*Y*-axes and a *Z*-axis linear actuator, the spatial system provides ample space for greater customizability. However, a specific user defined task dictates the design of a robust liquid handler, ultimately requiring careful planning and iterative improvement. A demonstration of the liquid handler running can be viewed in Video S2. A component breakdown and build guide for creating this specific handler is provided in a GitHub repository at https://github.com/GormleyLab/Pipette-Liquid-Handler.

### 3.2 Pen plotter liquid handler

For the remainder of this user guide, we consider the pen plotter liquid handler. In addition to the build guide, we provide an extended explanation of the low-level decisions related to its operation and production.

The integration of a finely tuned fluidic system becomes the major focus in a liquid handler using a prebuilt spatial system. A fluidic line was developed to produce the desired connection between the syringe pump and pen plotter to aspirate and dispense reagents. The developed configuration utilizes two parallel 1 mL syringes clamped into the syringe pump joined by a Y-assembly to a single tubing line ending with a needle assembly. The needle assembly is placed in a 3D printed holder on the *z*-axis linear actuator for withdrawal and dispensing by the pen plotter (Fig. 7). Two 1 mL syringes were chosen to leverage a smaller internal diameter to increase the reliability of dispensing at specified flow rates. However, larger syringes can be used for an increased storage capacity, albeit at the cost of lower dispensing accuracy and precision.

#### 3.2.1 Preliminary setup

Here, we review important aspects related to the setup of the pen plotter liquid handler.

##### 3.2.1.1 Fluidic line priming

Before the fluidic line can be used in the fluidic system, the line must be prepared. This is done by filling the line and syringes with an incompressible liquid (*i.e.* water), taking care to mitigate the number of air bubbles in the system. If the fluidic line exceeds the amount of volume present in the syringes, it may be advantageous to fully submerge the syringe in a container of the incompressible liquid with its connecting piece to fill the line without introducing unnecessary air bubbles. The loaded fluid transmits the forces generated by the syringe pump within the system to execute dispensing orders consistently. To eliminate interactions with the liquid already in the system, a deliberate air gap is formed to separate drawn fluids.

##### 3.2.1.2 Syringe pump fluidic calibration

Due to inaccuracies inherent in a pressure-driven syringe pump and variation in syringe sizes, it is necessary to scale the user-submitted volumes when dispensing. A scaling factor is determined by performing a calibration, which involves computing the ratio between actual and user-specified volumes for triplicate dispenses. When user-specified volumes are scaled by this factor, the liquid fluidic system dispenses volumes more accurately.

#### 3.2.2 Fluidic capabilities

The pen plotter liquid handler was designed to maximize accuracy and precision over the largest range of dispensing volumes possible while satisfying cost constraints, as described in Fig. S1. However, there is a clear lower limit for accurate volume transfers. At low volumes, it is measured to have higher dispensing error, likely due to start up effects of the syringe pump. This start up effect is present over the entire dispensing range as there is a consistent loss in energy due to compression at the air gap separating the incompressible fluid and the drawn reagent. However, this effect is remedied at higher volumes using the defined correcting factor.

### 3.3 Program

The software that provides programmable control of the liquid handler is key to developing automated workflows. The software must consider limitations of the spatial and fluidic systems to execute experiments of interest. Our software is built to work with the AxiDraw V3 pen plotter (now sold by Bantam Tools as NextDraw), Fusion 200X ChemyX syringe pump, and SpectraMax M2. The software was built using Python 3.12.0 and requires the ChemyX API, AxiDraw API and Molecular Devices SDK, as well as several packages for data analysis and interpretation like NumPy and Pandas. The program is organized into a modular structure to support flexibility and maintainability. At the top level, a 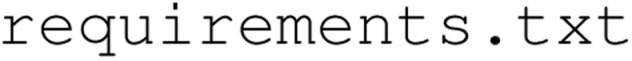 file defines all necessary dependencies installed in a virtual environment. The application is launched *via*
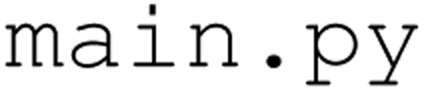
. The core functionality resides in the 
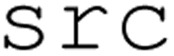
 directory, which includes five modules. The 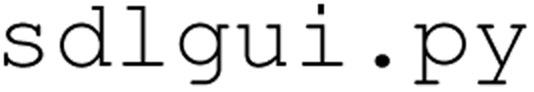 module contains all tkinter-based UI elements, including buttons and entry boxes used to configure experiments. The 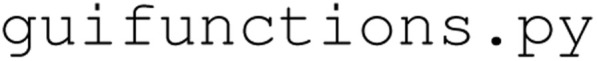 module is responsible for processing, organizing, and manipulating user input, and contains the functions that execute the experimental workflow. The 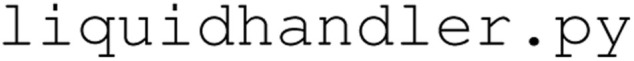 module controls the AxiDraw V3 and Fusion 200X syringe pump used for automated liquid handling. The 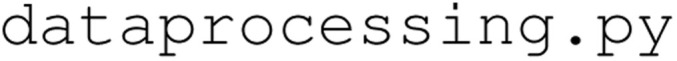 module handles data acquisition from the SpectraMax M2 and implements the ML and BO algorithms that enable self-driving functionality. Finally, 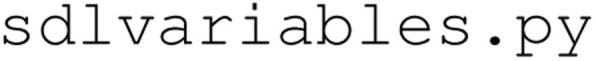 stores shared global variables such as directory paths and pandas DataFrames that are accessed and modified across the modules. The project also includes a data directory for storing raw and processed experimental data and a resources directory that contains static assets such as logos and icons used in the user interface.

When running the program, we provide a user interface that allows for the manual setup of experimental parameters. Configuring experiments begins in the ‘home’ tab, where a list of reagents is provided with corresponding concentrations. The order of the reagents determines the order of dispensing. When reagents are put into groups, the program selects one reagent from each group. Reagents that act as buffers must be selected as buffers in the dropdown menu and include a corresponding pH. Additional tabs can be added for different experiments. Currently, the enzyme assay tab is the only self-driven task that can be chosen. Under the enzyme assay tab, reagent bounds can be chosen in the format “([Lower Bound], [Upper Bound])”. The number of samples is the seed library size and must be chosen carefully to ensure the dispensing maximum is not exceeded. Finally, the desired enzymatic activity value is selected as the single-objective optimization target for the AL/BO pipeline. By providing example software, we hope to provide a starting point which users can add to or modify for their own experimental procedures.

## 4 SDL demonstration: enzyme assay optimization

In this section, we provide a representative example of our SDL in operation. Specifically, we apply the SDL pipeline to the optimization of an enzyme assay, a common objective in wet labs that can be challenging due to its dependence on many input parameters. We chose glucose oxidase (GOx) as the enzyme assay of interest due to its common use, colorimetric nature, and ability to integrate with our designed system. Within the AL framework, reagent concentrations are inputs, and enzymatic activity is the target property we seek to maximize. We discuss the development process step-by-step as a tool for improved understanding of the SDL pipeline.

### 4.1 Design

The GOx assay involves a two-step enzymatic reaction. The first step involves the oxidation of glucose to hydrogen peroxide, which is catalyzed by GOx. The second step involves the oxidation of 2,2′-azino-bis(3-ethylbenzothiazoline-6-sulfonic acid) (ABTS), which is catalyzed by horseradish peroxidase (HRP). Our target is a specified enzymatic activity of GOx, which can be measured by colorimetric analysis of the oxidized ABTS using a spectrophotometer.

To optimize the GOx assay, we must determine the viable space of input parameters. The input variables consist of concentrations of assay reagents, which includes GOx, HRP, glucose, and ABTS concentrations. Allowable ranges for these inputs must be chosen according to domain knowledge; for example, since our goal is to measure the enzymatic activity of GOx, it is necessary that HRP has a high enough concentration so that it is not the limiting reactant. We choose a GOx concentration range of 2.6 to 13 nM, while HRP has a concentration of 22.7 to 114 nM. ABTS and glucose were set at concentration ranges of 168.2 to 841 µM and 69.4 to 347 mM, respectively. Once we determine our input variable ranges, we generate a library of possible combinations of input variables. From this library, we employ the LHS space-filling algorithm (as discussed in Section 2.2) to generate the seed dataset. This results in an initial set of input parameters with a large coverage of the input space that can be used to initiate AL/BO.

### 4.2 Build and test

After determining a set of reagent concentrations to measure, we use the pen plotter liquid handler to measure enzyme activity. Determining a viable and reproducible protocol for running the enzyme assay on the pen plotter liquid handler requires substantial effort. For example, to measure enzymatic activity, we require kinetic sampling of the initial rate of change of absorbance (ΔOD) produced by the oxidation of ABTS to yield a blue-green product. Therefore, order of reagent addition is important; the reagent which initiates the reaction is added last to reduce time variance between wells. In this case, glucose drives the initial enzymatic event of the assay, so it is added last. Once the experimental procedure is determined, the program calculates and formats the dispensing protocol for the selected inputs, which is then executed autonomously by the pen plotter liquid handler. Leveraging automation minimizes human labor and reduces the potential for experimental errors.

Enzyme activities are measured by the SDL using an absorbance read method previously created using external Softmax software. We choose an initial seed dataset of sixteen reaction conditions, which was the maximum number of samples that our fluidic system could run in a single synthesis step. After data seeding, we acquire data in batches of six measurements, which was chosen as a reasonable compromise between time and resource consumption for our system. To provide the software enough time to collect kinetic data for a 96-well plate, the sampling rate is standardized for all iterations. We test samples using a 405 nm wavelength in fifteen-second intervals for two and a half minutes, permitting the measurement of (ΔOD) at standard time points. By using the pen plotter liquid handler described in Section 3, we can directly translate generated experimental designs into fully executed and characterized assays, providing high-quality data to train the surrogate model.

### 4.3 Learn

After measuring enzyme activities for the reagent concentrations chosen in the seed dataset, we train a surrogate model on the available data. We choose a GP model as the surrogate. We choose the GP kernel to be a summation of the Dot Product, RBF, and White kernels. We choose the Dot Product and RBF kernels due to their popularity; we choose the White kernel to capture noise in our data originating from formulation and testing. Kernel hyperparameters are chosen by performing a grid search, and we choose hyperparameters which maximizes *R*^2^ calculated using five-fold cross-validation. After training our GP surrogate model, we compute the EI acquisition function and identify the reagent concentrations which maximize EI. These reagent concentrations are identified, measured by the liquid handler, and the appropriate data is added to the dataset. This loop repeats until our stopping criterion is reached; here, we stop after four rounds of BO.

**Fig. 8:**
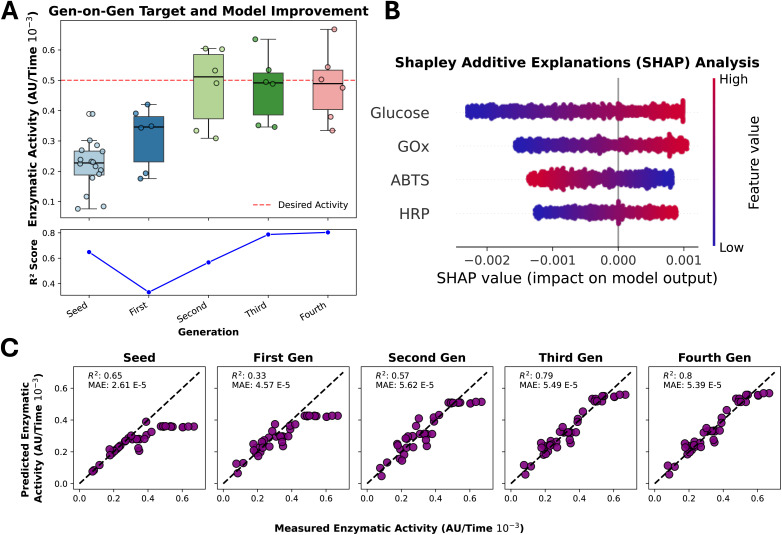
AL campaign performance and feature influence (A) formulation performance with respect to target enzymatic activity per generation overlaid with model performance per generation. Each formulation activity value is shown on a box and whisker plot over each iteration of the DBTL loop, with the target formulation value designated by a red dashed line. Performance per generation is shown by the line plot (Blue) of the quantitative metric of *R*^2^. (B) Shapley additive explanations (SHAP) analysis plot of GOx assay GPR model. The relative impact of each feature on model output (SHAP value) is displayed across the *x*-axis, and the relative magnitude of the feature value for each data point is color-coded across a gradient (red = high value, blue = low value). (C) Model performance displayed over each subsequent generation of the DBTL loop. Each diagonal black line shows a “perfect” model's prediction of the target feature, with values below the line representing an underprediction of the actual enzyme activity and values above the line representing an overprediction. Quantitative metrics of model performance (Gaussian process regressor *R*^2^ score and MAE) are listed in the top left corner of each graph.

Fig. 8A provides a summary of BO performance, reporting both the distribution of measured enzyme activities and prediction accuracies of surrogate models for each generation (*i.e.*, iteration). Fig. 8A shows a clear increase in the distribution of enzyme activities from round-to-round, with the primary increases occurring at rounds one and two. Such a distribution shift in the enzymatic activity of the seed data and later generations is indicative of a successful BO campaign; the SDL effectively biases recommendations in regions likely to maximize enzyme activity.

We also consider how surrogate model accuracy varies across the five rounds of data acquisition. Fig. 8A shows the cross-validation *R*^2^ score for the GP model for each generation. Initially, the surrogate model has relatively high predictive power, with an *R*^2^ = 0.65. The surrogate model experiences a decrease in accuracy after the first round of active learning, with an *R*^2^ = 0.33, but increases in accuracy for all subsequent generations, with a maximum accuracy of *R*^2^ = 0.80 by the final round. To better understand model accuracy at each generation, we analyze parity plots of predicted *vs.* measured enzymatic activity at each iteration, which we show in 8C. At early generations, the model struggles to make accurate predictions on reagent concentrations that result in high enzymatic activity. As the generations continue, high-performing predictions fall closer to the parity line and model accuracy increases. These results suggest that at early generations, model error is high due to inaccurate predictions for high-performing candidates. Model accuracy initially decreases as more high-performing candidates are added to the training set in the first round of BO. As the dataset size increases, the model improves its accuracy when predicting high-performing candidates. The overall increase in model accuracy and increased identification of high-performing reagent concentrations indicates a successful BO scheme.

An accurate surrogate model can be used to identify how changes in input parameters produce changes in experimental outcomes. Specifically, explainable AI (xAI) methods, such as Shapley Additive explanations (SHAP), provide a way to quantify the contribution of each reagent to model predictions of enzyme activity. In situations where the relationship between reagent and output is well-established, such analysis can assess whether the model has accurately captured the expected physical phenomena. 8B shows the results of applying SHAP analysis to our final surrogate model. The top two rows of 8B indicate that, according to the surrogate model, higher values of GOx and glucose concentrations contribute to increases in predicted enzymatic activity. This agrees with our physical intuition, since increasing concentrations of catalyst and substrate should increase reaction rates. The third row shows that higher concentrations of ABTS result in reductions in predicted enzyme activity; this agrees with prior work which shows that ABTS can act as an agonist at higher concentrations. The fourth row shows that concentrations of HRP have the least impact on predicted enzyme activities. Since HRP concentrations were specifically chosen to prevent HRP from being a limiting reactant, it is sensible that HRP concentrations would have a negligible impact on the resulting GOx assay. The results in 8B indicate that our model has accurately identified the expected physical relationship between reagent concentrations and enzymatic activity.

Due to the limitations of the device allowing for a maximum of 96 samples per experiment, it requires a manageable problem to solve before exhausting the resources of the system. The BO scheme was able to solve and understand the mechanisms of the assay within four generations of DBTL cycling after detecting sufficient convergence of model performance (*R*^2^ ≥ 0.8 and no significant improvement from previous generation). The optimization of GOx to achieve a certain enzymatic activity through iterative testing validates the SDL and BO scheme to educate and learn about SDL workflows.

## 5 Summary and perspectives

The goal of this perspective is to provide readers with a foundational understanding of AL principles in the context of developing self-driving systems capable of performing iterative experimentation. Our described system provides an open-source, customizable platform for automated liquid handling, reducing costs while maintaining precision and scalability for small-to medium-scale laboratory workflows. Through a representative study, we validated our low-cost automated liquid handler platform, providing readers with the tools and hardware necessary to apply SDLs to their own areas of expertise. We believe that the development of tutorials describing low-level component analysis of automation platforms and programming tasks shows noteworthy progress in the goal of democratizing user-developed automation infrastructure. By focusing on increased accessibility, there is a greater opportunity for accelerated adoption of SDLs as technical and financial barriers to entry continue to fall.

## Author contributions

A. P. M. and D. M. W. designed and validated low-cost liquid handler systems with build guides, designed and implemented user interface and program for liquid handling systems and wrote tutorial notebook 2. Q. M. G. and R. A. P. created tutorial notebook 1. A. P. M., D. M. W., Q. M. G., and R. A. P. wrote the initial draft. All authors revised the manuscript. M. A. W. and A. J. G. conceived the project and supervised the work. All authors contributed and approved the manuscript.

## Conflicts of interest

The authors declare no competing interests.

## Supplementary Material

DD-005-D5DD00525F-s001

DD-005-D5DD00525F-s002

DD-005-D5DD00525F-s003

## Data Availability

The datasets and code generated during this study, including experimental logs, build guides, device-control code, and Jupyter notebooks, are openly available *via* our GitHub repository (https://github.com/gormleylab/SDLGuide). A citable, archived version is available on Zenodo (https://doi.org/10.5281/zenodo.19190906). An easy-to-navigate project landing page can be found at https://gormleylab.github.io/SDLGuide/. Supplementary information (SI) is available. See DOI: https://doi.org/10.1039/d5dd00525f.
