# Supplementary material for: A user's guide to your first self-driving liquid handling lab
Source: Digit Discov. 2026 Mar 25;5(5):2028–41. doi: 10.1039/d5dd00525f (PMC13071554; doi:10.1039/d5dd00525f)
Supplement: DD-005-D5DD00525F-s001 [file DD-005-D5DD00525F-s001.pdf]

Supplementary Information (SI) for Digital Discovery.
This journal is © The Royal Society of Chemistry 2026

A User's Guide to Your First Self-Driving Liquid Handling Lab

Apostolos P. Maroulis,[†] Dylan M. Waynor,[†] Quinn M. Gallagher,[‡] Roshan A. Patel,[‡] Matthew Tamasi,[†] D. Christopher Radford,[†] Michael A. Webb,[*,‡] and Adam J. Gormley[*,†]

†Department of Biomedical Engineering, Rutgers, The State University of New Jersey, Piscataway, NJ 08854, USA

‡Department of Chemical and Biological Engineering, Princeton University, Princeton, NJ 08544, USA

E-mail: mawebb@princeton.edu; adam.gormley@rutgers.edu

Pressure Driven Liquid Handler Dispensing Calibration Sampling

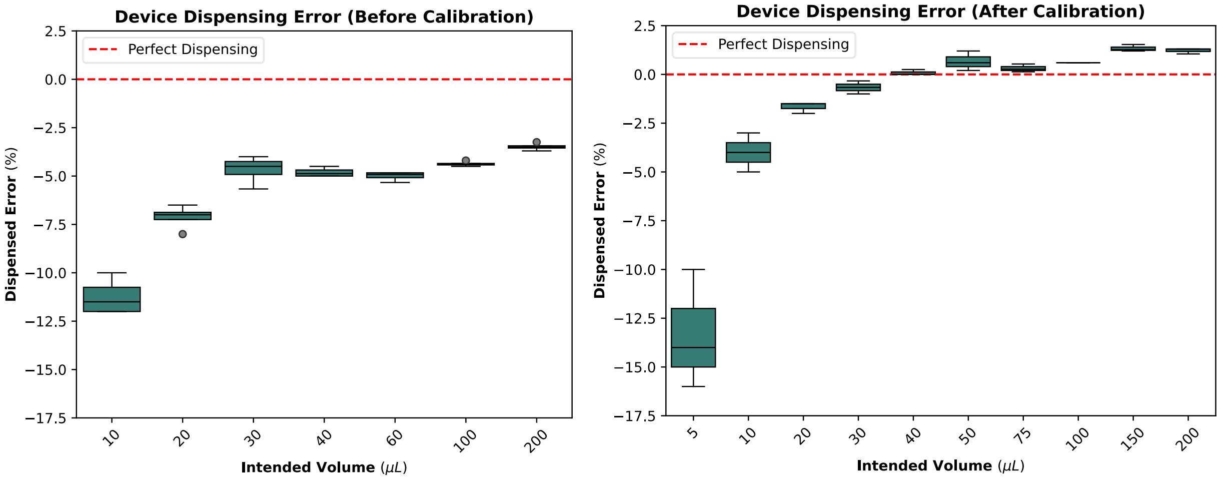

Figure S1. Pressure driven system dispensing error before and after calibration efforts. Red dotted line shows perfect dispensing.

Pen Plotter Liquid Handler Self Driving Lab

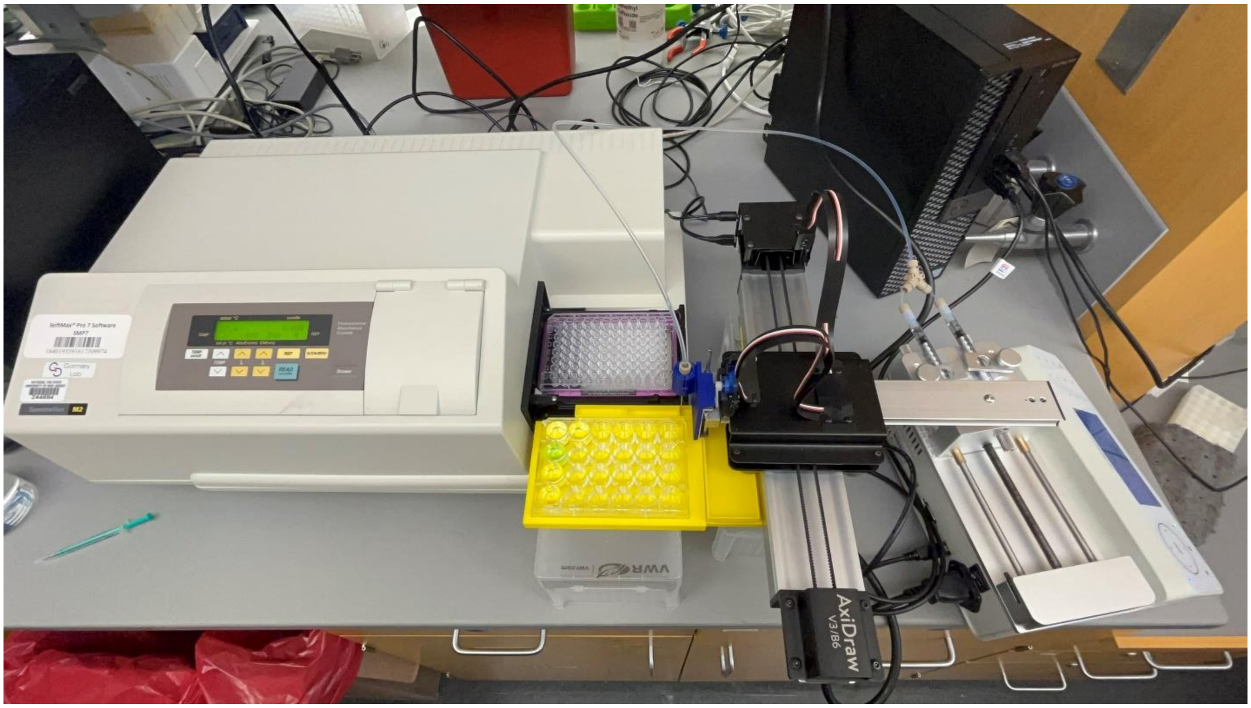

Figure S2. Pen plotter liquid handler integrated with a spectrophotometer.

Parameter Space Table

Overview of Design Space		
**Additive**	**Class**	**Concentration Range**
Phosphate Buffered Saline (PBS) (pH 7.4)	Buffer	0.1 M
Horseradish peroxidase (HRP)	Enzyme	22.7-114 nM
Glucose oxidase (GOx)	Enzyme	2.6 - 13 nM
Glucose	Substrate	69.4-347 mM
2,2'-azino-bis (3-ethylbenzothiazoline- 6-sulfonic acid) (ABTS)	Chromogenic substrate	168.2-841 μM

Table S1. Described design space used for GOx active learning campaign. Describes the name of the additive, class, and overall concentration ranges used.
